# Higher education privatization in Nursing in Brazil: profile, challenges and trends*

**DOI:** 10.1590/1518-8345.4725.3417

**Published:** 2021-06-28

**Authors:** Amanda Cavada Fehn, Thays dos Santos Guaraciaba Alves, Mario Roberto Dal Poz

**Affiliations:** 1 Organização Mundial de Saúde, Oficina Central de Enfermagem, Genebra, GE, Switzerland.; 2 Universidade do Estado do Rio de Janeiro, Instituto de Medicina Social, Rio de Janeiro, RJ, Brazil.

**Keywords:** Nursing Education, Nursing Statistics, Nursing Schools, Privatization, Nursing Economics, Brazil, Educação em Enfermagem, Estatísticas de Enfermagem, Escolas de Enfermagem, Privatização, Economia da Enfermagem, Brasil, Educación en Enfermería, Estadísticas de Enfermería, Facultades de Enfermería, Privatización, Economía de Enfermería, Brasil

## Abstract

**Objective:**

this study aims to analyze and characterize the movement of expansion of Nursing undergraduate courses in Brazil since the 1990s. The characteristics of this expansion are discussed, as well as the socio-political setting where such movement occurred, and the quality of education available based on the data collected.

**Method:**

this is a descriptive and cross-sectional study with a quali-quantitative approach, with the use of secondary databases.

**Results:**

an accelerated and disordered growth of Nursing undergraduate courses was identified, as well as the number of vacancies, especially due to the participation of the private sector, especially since the year 2000. Geographical inequalities in the distribution of these courses and vacancies were also identified.

**Conclusion:**

the strong expansion of higher education in Nursing, along with other health professions, resulted in the strengthening of private higher education institutions associated with economic groups, regional concentration, as well as the excessive offer of distance learning without adequate evaluation of its quality or repercussions.

## Abstract (pt)

**Objetivo:**

o presente estudo visa analisar e caracterizar o movimento de expansão dos cursos de graduação de enfermagem no Brasil a partir dos anos 90. Discutem-se as características desta expansão, o cenário sócio-político em que ocorreu tal movimento, bem como a qualidade do ensino disponível com base nos dados coletados.

**Método:**

trata-se de um estudo descritivo, transversal e de abordagem quali-quantitativa, com o uso de bases de dados secundárias.

**Resultados:**

foi identificado um crescimento acelerado e desordenado dos cursos de graduação em enfermagem, bem como do número de vagas, devido especialmente à participação do setor privado, em especial a partir do ano 2000. Desigualdades geográficas na distribuição desses cursos e vagas também foram identificadas.

**Conclusão:**

a forte expansão do ensino superior em enfermagem, ao lado de outras profissões de saúde, resultou no fortalecimento de instituições privadas de ensino superior associadas a grupos econômicos, na concentração regional, bem como na oferta excessiva da modalidade de ensino a distância sem avaliação adequada de sua qualidade ou repercussões.

## Abstract (es)

**Objetivo:**

el presente estudio tiene como objetivo analizar y caracterizar el movimiento de expansión de las carreras de grado en enfermería en Brasil a partir de la década del 90. Se discuten las características de esta expansión, el escenario sociopolítico en el que ocurrió este movimiento, así como la calidad de la educación disponible según los datos recopilados.

**Método:**

se trata de un estudio descriptivo, transversal con enfoque cualitativo y cuantitativo, utilizando bases de datos secundarias.

**Resultados:**

se identificó un crecimiento acelerado y desordenado de las carreras de licenciatura en enfermería, así como el número de cupos, debido fundamentalmente a la participación del sector privado, especialmente a partir del año 2000. También se identificaron desigualdades geográficas en la distribución de las carreras y los cupos.

**Conclusión:**

la fuerte expansión de la educación superior en enfermería, junto con otras profesiones de la salud, favoreció el fortalecimiento de las instituciones privadas de educación superior asociadas a grupos económicos, la concentración regional y la oferta excesiva de educación a distancia sin la correcta evaluación de calidad o de las repercusiones.

## Introduction

The global growth of Private Higher Education Institutions (PHEIs), especially those for profit in recent decades, has broadened the debate on the nature of public and private assets in higher education and especially on the role of the private sector and its impact on academic education. The educational system responds both to the demands of the health system and to the dynamics of the health labor market. In this sense, educational institutions are recognized as vital in the transformation of the health system and in economic and social development^(^ 1 ^)^.

The phenomenon of privatization in higher education in health has been characterized as a marked dynamic and accelerated growth in education in the 21^st^ century^(^ 2 ^)^. Its trends of expansion are especially related to the public policies, which influence, and even encourage, the increase of these institutions^(^ 3 ^)^.

In this study, the term “privatization” includes the expansion of private higher education institutions which offer Nursing degree courses, directed by individuals or companies, and no longer by the State. In this process, there is a tendency to perceive the student as a consumer and education as a product^(^ 4 ^)^. Privatization also presents as a characteristic a strong appeal of private HEI brands, competition, the exploitation of niche markets with socially prestigious professions, aggressive marketing, and profit-oriented pricing policies^(^ 5 ^)^.

In Brazil, the first Nursing school appeared in 1890, in Rio de Janeiro and, in 1939, the first private Nursing institution was created in São Paulo. Until the end of the 1950s there were only 33 Nursing courses in the country, of which 36% were of a private nature, in its immense majority confessional institutions^(^ 6 ^-^ 7 ^)^. In July 2019, the number of schools and actual vacancies were 1,348 and 197,995, respectively.

While the increase in the number of schools and vacancies can be considered positive, since it has increased access to higher education and improved the proportion of professionals *per* inhabitant, on the other hand, it has produced inequalities in the distribution of institutions and questions on the quality of education. Additionally, Nursing training in Brazil started to strongly contemplate the Education at Distance (EaD) modality, with 82.000 vacancies offered by 9 Higher Education Institutions (HEIs), all private^(^ 6 ^)^.

Nursing is the largest professional category in the health sector. Recent data from the World Health Organization (WHO) indicate a growth of 4.7 million professionals in the period from 2013 to 2018, currently corresponding to approximately 59% of the workforce in the sector worldwide^(^ 8 ^)^. However, 80% of these professionals are located in countries with half the world’s population, a very unequal distribution especially in the poorest and low- and mid-income countries. Despite this growth, until 2030 a shortage of 5.7 million Nursing professionals is estimated, especially in countries with the highest burden of diseases^(^ 8 ^)^.

There is no evidence on how the increased number of vacancies and schools could solve the problem related to the lack of health professionals^(^ 9 ^)^. Recent WHO analyses indicate the need for growth in the mean number of Nursing graduates around 8% per year, but stress the importance that this expansion must be linked to greater employability, strategies for the retention of these professionals, development of competencies and skills associated with the needs of health systems, and strengthening governance and leadership in Nursing^(^ 8 ^)^.

Currently, in Brazil there are 1,241 Nursing courses in the face-to-face modality under activity, that offer 193,217 vacancies, the immense majority being in private HEIs, whose participation is of 87.8% in the offer of courses. The increase in the participation of the private sector, inherent to the global trends of the economic model, in the offer of services and in health education, also created opportunities in the measures that aimed at the expansion of the Universal Coverage of Health and the flexibilization of laws and norms regarding the participation of private and international capital, besides reducing the inefficiency of regulatory structures^(^ 9 ^)^.

The understanding of workforce training is intrinsically linked to economic development and has important social, economic and political consequences. It is difficult to assume that large economies reach advanced stages of development without high levels of innovation and quality in education, for example, which can be considered basic components for placing a country at the forefront of the “information society”^(^ 10 ^)^. In this sense, it is crucial to understand how universities operate in the process of training human resources in market terms, since this reality does not always appear in the public description of their function and often receives a negative connotation^(^ 11 ^)^. Moreover, it is urgent to understand the factors affecting the size of the future health workforce to plan and propose more effective actions adjusted to each reality^(^ 12 ^)^.

Thus, this article intends to describe, analyze and characterize the expansion movement of Nursing undergraduate courses in Brazil, emphasizing the participation of the private sector, its role, impact and trends in Nursing training in the country.

## Method

In Brazil, in accordance with the Law No. 7,498/86^(^ 13 ^)^, nurses, nursing assistants and technicians, obstetricians and midwives are defined as Nursing professionals. Training takes between one and five years, varying according to the degree and the title. In this article, only the information referring to the professional qualified in higher level Nursing courses is analyzed, whose undergraduation varies from 4 to 5 years.

It is a study of the descriptive type, cross-sectional and with a quali-quantitative approach, whose analysis setting is Brazil. The quantitative data of this study were collected in the period going from July 2019 to May 2020. The population of this study is the higher education institutions with Nursing training courses, selected with the following criteria: i) higher education institutions with undergraduate Nursing courses; ii) the administrative category of IES; iii) active course; and iv) teaching modality, if face-to-face or distance learning. All the institutions with undergraduate courses in activity were selected as Participants.

The following variables of the study were selected: number of active Nursing courses, modality of the activity, if face-to-face or at distance, administrative category of the institution that offers the graduation course (if public or private), number of authorized vacancies, and the geographic location of the course or of the institution that offers it. As the variables were easily located, it was not necessary to use any particular instrument for data collection.

Data collection was carried out at the e-MEC websites, the Anísio Teixeira National Research Institute (INEP) and the Workstation of the Observatory Network of Human Resources in Health of the Institute of Social Medicine of the State University of Rio de Janeiro (ObservaRH-IMS/UERJ). Regarding data treatment and analysis, we highlight that, at the e-MEC website, we obtained the listed data referring to the period in force and effect, and that it was not possible to access data from previous years, which were obtained from the ObservaRH-IMS/UERJ database. The microdata from the 2017 and 2018 Higher Education Census were obtained from the Anísio Teixeira National Research Institute (INEP) website. The entire collection was conducted by a single researcher, with supervision and review by another researcher. Subsequently, the data were compiled and organized into a table and graphs, using Office Microsoft Excel resources.

The literature review was carried out in the PubMed, SciELO and Google Academic databases, in such a way to become aware of the existing production of the education of nurses and of the expansion of private higher education in this area. For this, the following search terms were used: *nursing education, nursing schools, private and privatization*. Relevant articles published since the year 2000, in English, Portuguese and Spanish, dealing with similar research studies and/or the historical context of the development and expansion of Nursing undergraduate courses were considered. The search of the texts was made by a researcher, with the participation of the other researchers in the analysis, in a qualitative manner, with emphasis on the content of the selected articles, in order to complement the information of the research, as well as to compare them to other studies. Finally, regarding the ethical aspects, this study waives the approval of any Ethics and Research Committee.

## Results

Changes in the education systems have expanded permeability to the private sector^(^ 14 ^)^. By permeability we can understand the effects of the meeting or overlapping of the State and the private sector in the various stages of production and delivery of public policies^(^ 15 ^)^. The effects can assume different outlines depending on the relational pattern established at a given time, under certain historical, social, political and economic conditions^(^ 15 ^)^.

The purchase and sale of services is no stranger to the state activity; however, the speed and the characteristics of privatization in the last decades had much proper aspects, especially in the educational sector. In this sense, there are no services exempted from the participation of the private sector; there are those where the private sector operates with less or very little expression^(^ 16 ^)^.

This section initially presents the profile of the privatization process in Brazil, highlighting some elements of the historical context and its characteristics. The following describes the growth trend of the private sector over the past few years and its geographical distribution across states and regions of the country.

### The privatization of higher education in Brazil

As far as Brazilian higher education is concerned and from a legal point of view, the State can and has transferred resources to private HEIs. In the case of the public policies that influence and even contribute to the expansion of private higher education institutions, there is a tendency to increase the expressiveness of the private sector in the health sector globally^(^ 3 ^)^.

The expansion of Brazilian higher education covers four categories: 1) expansion of vacancies, enrollments and courses in the federal HEIs by the Union, which can be observed both in the multiplication of *campi* of existing federal institutions, such as in the increase of the number of institutions or through restructuring programs, such as the Support Program for Restructuring and Expansion Plans of Federal Universities (Reuni); 2) expansion of post-graduation and knowledge entrepreneurship; 3) intense diversification of courses, institutions and teaching modalities, especially distance learning (EaD); and 4) expressive increase of private for-profit HEIs^(^ 17 ^)^.

From 1960 to 1970, a lucrative sub-sector of higher education businesses was created, when the establishments were still considered formally as non-profit institutions^(^ 18 ^)^. Another interesting aspect is that, although political scenarios have alternated between different political groups and parties with supposedly different ideals of conformation, orientation and militancy, their leaders have maintained a favorable direction for the expansion of private HEIs^(^ 19 ^)^.

The 1996 Guidelines and Bases Law (*Lei de Diretrizes e Bases, LDB*) is considered by most authors to be the basis of the privatization process of higher education in Brazil. However, the participation of private for-profit HEIs, explicitly or not, already existed since the 1960s^(^ 7 ^,^ 19 ^-^ 20 ^)^.

In the 2000s, the private HEI market was stimulated by public policies associated with public financing mechanisms, which favored the expansion of higher education. In the last decade, the growth, consolidation and merging of controlling groups of HEIs enabled the transformation of numerous educational companies into business conglomerates, with the possibility of new organizational formats, such as the establishment of oligopolies^(^ 21 ^)^.

Linked to the phenomenon of privatization is the commodification of Brazilian higher education, where the expansion of for-profit institutions and the adoption of market strategies oriented to funding, oligopolization and internationalization stands out. These transformations in the Brazilian educational model open the way to the understanding of education as a commercialized product, object of profit and benefits to the owners and shareholders. The interest for this branch is evidenced by the diversification of the “products”, such as the offer of face-to-face and distance courses, the production of didactic materials, the presence of business consulting and the strengthening of educational marketing, among others^(^ 22 ^)^.

### The growth trend in Nursing schools in Brazil

Until the end of the 1940s there were 20 Nursing courses in the country, four in private HEIs. Since 1949, the Brazilian State stimulated the creation of new schools, by making the Nursing course compulsory in all the universities or headquarters of medical schools^(^ 23 ^)^. Law No. 775 of 1949 signaled a strong bond between Nursing education and the labor market of the time and, therefore, was centered on the clinical model intrinsic to the enlargement of hospitals^(^ 24 ^)^. Only in the 1950s, 14 new Nursing schools were created, eight of them private institutions (57%).

**Figure 1:**
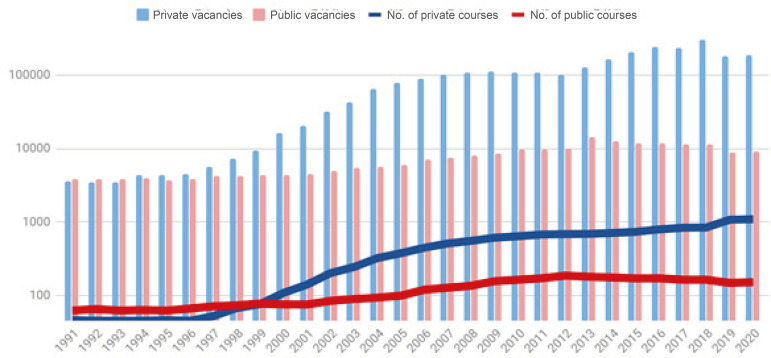
Evolution of Nursing courses and vacancies, 1991-2020. Brazil

After the 1968 University Reform, the Nursing degree finds again fertile ground for expansion. If in the 1960s only three new courses were created, and the 1970s saw the appearance of 39 new courses, 28 in public HEIs and 11 in private institutions. From 1980 to 1990, 62 Nursing schools were created, 42 in public institutions. The participation of private HEIs remained stable until the 1990s, when the enactment of LDB in 1996 promoted an alignment of the country’s educational policies with flexibilization, liberalization, diversification of financing, and redefinition of the role of the State in education. So it is from the late 1990s that growth in the private sector surpassed that of the public in the supply of courses and Nursing positions, as shown in Figure 1.

The public sector held more than half of the enrollment until 1998 but, in the following year (1999), private course enrollment represented 54.5% of the total in the country, continuing to grow over the years. Several factors contributed to the substantial expansion of Nursing courses and vacancies in the country and, consequently, in the number of graduates: i) legal and normative documents related to the allocation of Nursing professionals in services; ii) labor market flexibility; iii) public policies that corroborated the expansion of the labor market in the health sector; and iv) educational policies that make HEIs more tolerant and autonomous^(^ 25 ^)^.

**Figure 2:**
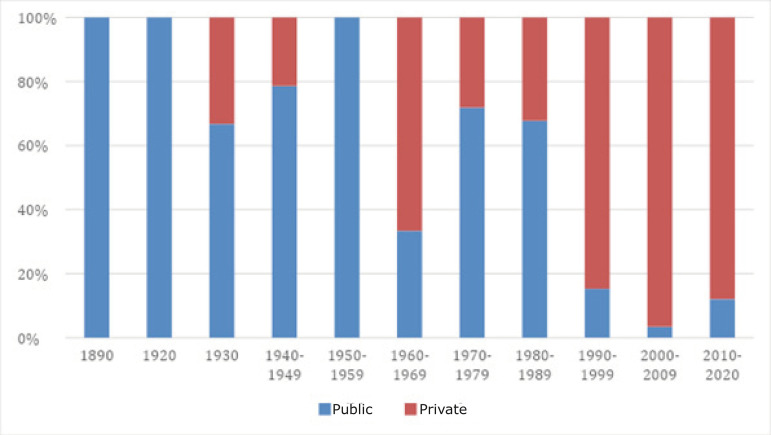
Percentage distribution for the administrative category of the Nursing courses in Brazil, between 1890 and 2020. Brazil

Between 1991 and 2004, the number of available vacancies increased tenfold, from 7,460 to 70,400, of which only 8% were in the public sector. The number of active courses in this period also increased expressively, going from 108 in 1991 to 415 in 2004, with a total increase of 291,5%, confirming the leading role of the private sector with a growth of nearly 616%. The LDB contributed strongly to this expansion, because in the period from 1991 to 1996, for example, the growth percentage of higher education in Nursing was only 4.71%, while between 1996 and 2004 it was approximately 287%. The same trend was noticed in the following years. In 2003 there were 243 Nursing degree courses offered by the private sector and 89 by the public; in 2004, these numbers were 322 and 93, respectively. The vacancies offered in 2003 totaled 41,937 in the private sector and 5,410 in the public sector; in the following year, the numbers jumped to 64,803 and 5,597, respectively, an increase of 154.5% of vacancies in the private sector. Figure 2 presents the proportional distribution of the administrative category of the Nursing courses in the period considered.

However, although the number of vacancies for Nursing undergraduate students has increased considerably, the number of graduates has not followed the same evolution; on average, the evasion percentage of Nursing vacancies is historically higher in private courses than in public courses and corresponds to 38% in public institutions and to 62% in private ones^(^ 26 ^)^. The worst percentages of vacancies were recorded in the years 2002, 2003 and 2004, with the equivalent of 18% in the private sector.

In 2006, the first distance Nursing courses were created. In 2019, 130 courses were offered by 9 institutions, distributed in 14 poles, totaling 82.000 vacancies available in the EaD modality. Today, this modality represents 28% of the total vacancies offered for Nursing under-graduation in Brazil. In the private sector, compared to the face-to-face modality, distance learning represents 30,8% of the vacancies offered.

### Distribution trend of the Nursing schools in Brazil

The growth in the number of courses has not been uniform throughout the country’s regions, presenting inequalities between states and cities and concentrations mainly in the capitals and metropolitan regions, where, in general, there is a higher Gross Domestic Product (GDP) and, consequently, greater economic power and greater concentration of income of the population, in addition to a previous installed capacity, both of human resources and of physical structure. The largest fraction of public investment has also occurred in these locations.

The Southeast region, followed by the South, remained the epicenter for expanding courses and vacancies. However, considering the proportion of expansion in the period from 1991 to 2004, it was the Midwest that had the most expressive increase, 480%, although in absolute figures the number of schools (n=29 in 2004) was much lower than in the Southeast and South regions (n=220 and n=84, respectively, for the year 2004). Table 1 shows the current geographic distribution of courses and vacancies in Brazil.

Regarding the enrollments, in 2018, the private institutions received 91.5%, most of them distributed throughout the Southeast region (37.8%), and followed by the Northeast region (31.1%). The Midwest and North regions had an index of just over 10% (10.9% and 10.5%, respectively) and the South had a lower enrollment rate (9.7%). The Southeast region stands out for having the highest enrollment rates in recent years, as seen in Table 1.

**Table 1:** Distribution of Nursing undergraduate courses and vacancies in Brazil, in 2020

Region	State	Public		Private		Total vacancies
		Courses	Vacancies	Courses	Vacancies	
North	Acre	2	60	3	630	690
	Amapá	3	150	5	760	910
	Amazonas	4	246	9	4.205	4.451
	Pará	6	286	27	4.450	4.736
	Rondônia	1	30	19	2.180	2.210
	Roraima	2	180	4	700	880
	Tocantins	2	400	9	1.080	1.480
	***Regional Total***	**20**	**1,352**	**76**	**14.005**	**15.357**
Northeast	Alagoas	3	140	16	2.822	2.962
	Bahia	9	476	83	13.580	14.056
	Ceará	6	410	47	7.463	7.873
	Maranhão	11	485	25	3.776	4.261
	Paraíba	5	410	17	3.580	3.990
	Pernambuco	7	435	50	9.974	10.409
	Piauí	8	430	19	2.605	3.035
	**Rio Grande do Norte**	**5**	**232**	**17**	**3.125**	**3.357**
	Sergipe	3	145	16	3.370	3.515
	***Regional Total***	57	3,163	290	50.295	53.458
Southeast	Espírito Santo	2	110	23	2.390	2.500
	Minas Gerais	12	862	122	14.910	15.772
	Rio de Janeiro	7	598	88	17.089	17.687
	São Paulo	12	688	230	49.188	49.876
	***Regional Total***	**33**	**2,258**	**463**	**83.577**	**85.835**
South	Paraná	11	449	56	6.750	7.199
	Rio Grande do Sul	7	447	49	5.710	6.157
	Santa Catarina	4	199	41	4.094	4.293
	***Regional Total***	**22**	**1,095**	**146**	**16.554**	**17.649**
Midwest	Federal District	3	260	31	7.068	7.328
	Goiás	5	190	40	6.275	6.465
	Mato Grosso	7	410	28	3.825	4.235
	Mato Grosso do Sul	4	190	16	2.700	2.890
	***Regional Total***	**19**	**1,050**	**115**	**19.868**	**20.918**
National Total		151	8,918	1.090	184.299	193.217

Source: e_Mec, 2020

Nine IES offer nursing courses in the modality EaD, with poles in almost all the Brazilian states. Two HEIs based in the state of São Paulo concentrate almost 80% of all vacancies offered, as observed in Table 2.

**Table 2:** Vacancies in distance nursing courses by IES. Brazil, 2020

HEI	ACRONYM	N. VACANCIES
Universidade Paulista	UNIP	47.880
Universidade Anhanguera	UNIDERP	16.800
Universidade Pitágoras	UNOPAR	8.000
Centro Universitário Planalto do Distrito Federal	UNIPLAN	4.260
Centro Universitário Mauricio de Nassau	UNINASSAU	2.000
Centro Universitário Braz Cubas	BRAZCUBAS	2.000
Universidade Potiguar	UNP	600
Centro Universitário Claretiano	CEUCLAR	400
Universidade Estácio de Sá	UNESA	60
TOTAL	-	82.000

Source: e_Mec, 2020

Note: In September 2020, the UNIP was authorized to increase from 47,880 to 73,260 its annual vacancies

## Discussion

Training in health is a vital component for the sustainability and effective functioning of any health system. The responsibilities and roles of nurses as clinical professionals, leaders, policy makers, researchers, scientists and professors are fundamental to the effective and practical functioning of education.

The trend towards privatization of health education seems to have a global dimension. In China, with government encouragement, private institutions for the education of health professionals developed rapidly, exceeding what public institutions were able to offer, but with unbalanced geographical distribution and questions on technical and educational capacity^(^ 27 ^)^. In Bahrain, Middle East, there is a strong incentive for students to enter the private sector, where they are offered a written guarantee of employment in the private sphere, for when they graduate^(^ 28 ^)^.

In Thailand in 2006, 19.6% of all the nurses trained in the country were graduates from private institutions; in 2010, this percentage rose to 24.1%, confirming the importance of the private sector in the training of these professionals^(^ 29 ^)^. Data from 2010 already signaled a large participation of the private sector in Kenya, with 51%^(^ 35 ^)^ of 68 Nursing EAD courses^(^ 29 ^-^ 30 ^)^. In India, in this same year, 88% of all the Nursing education institutions were private, producing 95% of the total Nursing workforce. In 2017, the total of private institutions already reached 90.3%. In Saudi Arabia, in 2017, of the 39 HEIs, 13 (33%) are private^(^ 31 ^)^.

The participation of the private sector in Nursing qualification around the world is very expressive and raises questions, which in general orbit around four topics: i) the contribution of the private sector in the training of Nursing professionals; ii) concentration/distribution; iii) the trend to also be linked to the private sector; and iv) teaching quality^(^ 9 ^)^.

In Brazil, this expansion movement can be associated with two factors: a reduction in funding in the public sector and a flexibilization and relaxation of regulations, including the emergence and increase of distance learning courses, which brings to discussion the component of quality of training.

Despite the distribution/concentration of the Nursing schools in the country, it is clear that the argument that the expansion of the private sector would serve to fill the gaps in the public sector corresponds to an opportunity for market expansion. This urban-centered pattern of distribution of private HEIs is also perceived in India, Kenya, South Africa and Thailand^(^ 29 ^)^, for example, and is also identified for medical schools^(^ 32 ^)^. Similarly, in Brazil private hospitals are concentrated in cities with better economies and so are private medical schools, most of them located in the Southeast (41.4%), especially in São Paulo and Minas Gerais, where most of the private hospitals are for-profit (57.8%)^(^ 33 ^)^. The concern with the imbalance in the geographical distribution also occurs in China, where 97 private HEIs are located in Western and Central China, economically more advantageous regions, while only 26 are in Eastern China^(^ 27 ^)^.

The disparities indicate how the interaction between the education system and the health system has been mediated by the Nursing labor market, taking into account the challenges of governance and regulation. The results revealed imbalances and critical mismatches in the demand and supply of nurses, especially in locations outside the major urban centers. In India, for example, only 9% of the Nursing schools are in locations with shortages of such professionals^(^ 29 ^)^. Although the supply of nurses has grown, it coexists with low rates of absorption of these professionals by the public sector, often due to recruitment inefficiencies or to poor working conditions. A similar setting has been described in Kenya and India, where the imbalance between production (supply) and labor market absorption (demand) has strongly favored the migration of nurses in these countries (20% and 25% of the nurses trained by the private sector in Kenya and India, respectively)^(^ 29 ^)^. Recent analyses of the WHO projections estimate that the effective global demand for nurses would increase by nearly 33% between 2019 and 2039, yet more than 220,000 professionals would not be absorbed either by the public sector, nor by the public sector^(^ 8 ^)^.

Although Brazil is not traditionally a “Nursing labor exporter” country, the growing trend in the number of schools and the high supply of vacancies, coupled with the inability to employ these professionals, suggest a powerful space for migration. In the richest countries, the existence not only of a workforce mostly older and close to retirement, but also of international recruitment companies that, besides subsidizing the language course, offer good employment conditions and social benefits, has increased the interest in Nursing professionals from poor and low- and mid-income countries (PLMICs).

At the same time, contexts of economic recession, widely seen in these countries, affect all sectors of the economy, including health professionals. Being the largest professional group in the health sector, nurses are strongly affected by the attempts to balance the budget, whose simplest and quickest alternative for adjustment is the reduction in the supply of workers^(^ 34 ^)^. Another aspect is the reduction or freezing of the Nursing wages, increasing the number of worked hours and/or of multiple jobs in searching to guarantee personal/family income^(^ 34 ^)^. In this context, the possibility of migrating abroad with the guarantee of a work contract can indicate a trend in the Nursing labor market. In the Philippines, for example, most private nursing schools have the participation of international groups, especially British and European, which contribute financially to the opening and operation of these institutions, in addition to drawing up the guidelines and curriculum guidance for the training of these professionals, so that they are compatible and equivalent to the standards required in their countries, strongly encouraging the migration of this workforce.

The exponential growth of the private sector alerts to the lack of qualifications of the teaching staff, which in general, in these institutions, presents higher turnover and less rigorous and more flexible criteria for the selection of professors. Another important point is that the increase in the number of schools increases the competitiveness of the most qualified university professors not only among private HEIs, but also with public institutions^(^ 11 ^)^. Also in the field of teaching quality evaluation, it is worth highlighting the existence of a gap in studies on the quality of the teaching staff in public and private educational institutions and the comparative percentage of physicians and masters, for example. It is known, however, that the vast majority of private HEIs in Brazil do not direct the curriculum to research and, generally, the leading role in the scientific field of research is centered on the public institutions.

Internationally, some studies suggest that public institutions, in general, are in a better position to respond to the countries’ FTS^(^ 34 ^)^ requirements, in terms of aligning student admissions and training programs with changes in the sociodemographic profile and population needs, maintaining health standards, establishing training schools in rural areas, and collaborating with the public health system, as internship settings during graduation^(^ 9 ^,^ 33 ^)^. However, more current studies are urgently needed.

The absence of an effective regulation mechanism by the State in Brazilian private higher education can be considered an incentive for expanding this sector^(^ 11 ^,^ 35 ^)^. Although the regulation of higher education is monitored by the Ministry of Education, this institution lacks mechanisms and tools more adjusted to the reality of the expressive quantity of HEIs and, in the specific case of Nursing, of the massive number of vacancies offered, especially in the at-distance modality.

Thus, although the present study presents some limitations and difficulties, derived from the use of different nomenclatures and modality of courses in the area and, despite the great amount of collected data, the analysis, paradoxically, is still exploratory. Due to the inconsistencies and knowledge gaps on these processes, it substantially contributes to the scientific knowledge of Nursing research in the area of management and evaluation of health education, whose production between the years 2006 and 2015 was limited to 11%. At the same time, this study seeks to broaden and enhance the discussion of the health workforce through the labor market lens, proposing an analysis on the growth of the private education sector and its impact on the health systems.

## Conclusion

This study examines three major questions on Nursing schools: The first relates to the strong expansion of higher education in Nursing alongside other health professions, which has resulted in the strengthening of private higher education institutions associated with economic groups and regional concentration. The second refers to the geographical distribution of the courses and vacancies, which is unequal in the country. And finally, the existence of the distance learning modality. It is still a controversial method and very questioned in courses in the health care area, since the training does not do without personal interaction, especially for the end-object of the Nursing exercise, that is, care. Concerns on the quality relative to the expressive increase in the number of courses and vacancies and the quality of Nursing training and of other health professions are fundamental for an effective planning of the health workforce.

The growing number of schools and vacancies does not seem to be linked to articulated strategies to solve the problem of the growing demand for a sufficient and qualified health workforce. This situation is similar to that of many developing countries that have failed to establish an organic relationship between multi-professional education and the health system. Innovative strategies to address this issue must be developed to contribute to achieving universal health coverage and access.

In the current context of the new coronavirus pandemic (COVID-19), distance learning has become a priority in the HEI agenda, even without much information on its development or its impact on the quality of training of the professionals and on the health labor market, except for the tendency to concentrate the educational offer in a few HEIs. This is a necessary and urgent research agenda in the context of teaching health professions.

The study sought to demonstrate the relevance and scope of the privatization process for Nursing education, reflecting on the implications of this process in meeting the demands of the health system in Brazil.

Furthermore, it is expected to contribute to the development of a more practical and participatory research agenda involving different actors from the government, academic settings, civil society, private sector and associations, among others, as well as to the process of evidence-based decision-making and political choices.
